# Memorandum for the Information of Persons Desirous of Entering the Medical Corps of the Army

**Published:** 1866-09

**Authors:** 


*Memorandum for the Information of Persons desirous of entering the Medical Corps of the Army.*—[Extracts from laws of the United States.] Act of Congress approved July, 1866.

"Sec. 17. *And be it further enacted*, That the Medical Department of the Army shall hereafter consist of one Surgeon General. * * * One Assistant Surgeon General. * * * One Chief Medical Purveyor and four Assistant Medical Purveyors. * * * Sixty Surgeons, with the rank, pay and emoluments of Majors of Cavalry. One hundred and fifty Assistant Surgeons, with the rank, pay and emoluments of First Lieutenants of Cavalry, for the first three years' service, and with the rank, pay and emoluments of Captains of Cavalry after three years' service, * * * and all the original vacancies in the grade of Assistant Surgeons shall be filled by selection by examination."

The number of vacancies now existing in the Medical Corps of the U. S. Army is sixty, forty-six of which are original vacancies created by the Act of Congress approved July 28, 1866, as quoted above.

All candidates for appointments in the Medical Corps, must apply to the Surgeon General, U. S. Army, for an invitation to appear before the Medical Examining Board. The application must be in the hand writing of the candidate, stating age and birth-place, and be accompanied by testimonials from Professors of the College in which he graduated, or from other physicians of good repute. If the candidate has been in the medical service of the army during the war, the fact should be stated, together with his former rank, and time and place of service, and testimonials as to qualifications and character from the officers with whom he has served should also be forwarded.

Candidates must be graduates of some regular medical college, proof of which must be submitted to the Board before examination.

